# Workshop on Measurement Needs for Local-Structure Determination in Inorganic Materials

**DOI:** 10.6028/jres.113.026

**Published:** 2008-12-01

**Authors:** Igor Levin, Terrell Vanderah

**Affiliations:** National Institute of Standards and Technology, Gaithersburg MD 20899

**Keywords:** diffraction, local structure, measurements, microscopy, nanostructure, spectroscopy

## Abstract

The functional responses (e.g., dielectric, magnetic, catalytic, etc.) of many industrially-relevant materials are controlled by their *local structure*—a term that refers to the atomic arrangements on a scale ranging from atomic (sub-nanometer) to several nanometers. Thus, accurate knowledge of local structure is central to understanding the properties of nanostructured materials, thereby placing the problem of determining atomic positions on the nanoscale—the so-called “nanostructure problem”—at the center of modern materials development. Today, multiple experimental techniques exist for probing local atomic arrangements; nonetheless, finding accurate comprehensive, and robust structural solutions for the nanostructured materials still remains a formidable challenge because any one of these methods yields only a partial view of the local structure. The primary goal of this 2-day NIST-sponsored workshop was to bring together experts in the key experimental and theoretical areas relevant to local-structure determination to devise a strategy for the collaborative effort required to develop a comprehensive measurement solution on the local scale. The participants unanimously agreed that solving the nanostructure problem—an ultimate *frontier* in materials characterization—necessitates a coordinated interdisciplinary effort that transcends the existing capabilities of any single institution, including national laboratories, centers, and user facilities. The discussions converged on an *institute* dedicated to local structure determination as the most viable organizational platform for successfully addressing the nanostructure problem. The proposed “institute” would provide an intellectual infrastructure for local structure determination by (1) developing and maintaining relevant computer software integrated in an open-source global optimization framework (Fig. 2), (2) connecting industrial and academic users with experts in measurement techniques, (3) developing and maintaining pertinent databases, and (4) providing necessary education and training.

## 1. Introduction

The functional responses (e.g., dielectric, magnetic, catalytic, etc.) of many industrially-relevant materials are controlled by their *local structure*—a term that refers to the atomic arrangements on a scale ranging from atomic (sub-nanometer) to several nanometers. Examples include nanostructured materials having atomic order limited by the nanoscale dimensions of their constituent particles or layers (e.g., nanoparticles, mesoporous materials, nanometer-thick layers in thin-film architectures) and nanostructured bulk materials exhibiting deviations from the average periodicity arising from local chemical and/or displacive order (e.g., dielectrics, relaxor ferroelectrics, thermoelectrics). The existing or envisioned applications for these materials impact the entire industrial spectrum including the multi-billion electronic, energy, automotive, and health sectors. The 2009 budget request for the U.S. National Nanotechnology Initiative alone is $1.5 billion to promote understanding of the unique phenomena and processes at the nanometer scale, and to expedite the use of this knowledge to advance practical applications. Accurate knowledge of local structure is critical for understanding the properties of nanostructured materials, thereby placing the problem of determining atomic positions on the nanoscale—the so-called “nanostructure problem”—at the center of modern materials development.

Unfortunately, traditional structure-solving approaches that assume long-range structural periodicity and rely upon Bragg reflections observed by x-ray/neutron diffraction methods fail on the local scale. Today, multiple experimental techniques exist for probing local atomic arrangements. A partial list includes various spectroscopic tools (e.g., based on x-ray absorption, Raman, nuclear magnetic resonance), rapidly emerging total x-ray/neutron scattering, and a suite of diffraction and imaging techniques implemented in modern transmission electron microscopes. Recent revolutionary advances in synchrotron, neutron, and electron microscopy instrumentation radically expanded the technical capabilities of many of these methods by providing critical high-energy/high-brightness probes. At the same time, modern theoretical calculations, especially those based on first principles, have become increasingly successful in predicting local atomic arrangements and their associated measurement responses (e.g., diffuse x-ray scattering or Raman spectra). Nonetheless, finding accurate comprehensive, and robust structural solutions for nanostructured materials still remains a formidable challenge because any one of the existing methods yields only a partial view of the local structure. The problem is further complicated by the substantial fractions of atoms in these materials associated with surfaces (external or internal). Additionally, the information is often needed at several length scales (e.g., from < 1 nm to 10 nm). A recent review article in Science [1] proposed a plausible path for solving the local-structure problem by integrating complementary information from multiple measurement techniques and theory into a global structure optimization framework that could be used freely by experimentalists and theorists alike. The article emphasized that the development of this framework requires a coherent focused interdisciplinary effort, which is currently missing.

## 2. Objectives and Description of the Workshop

**Fig. 1:**
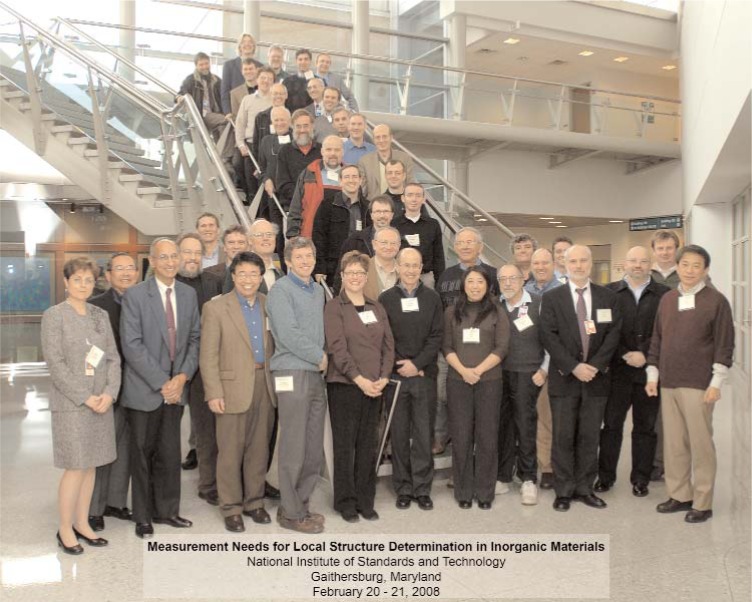
Group photo of workshop participants.

The primary goal of this 2-day workshop was to bring together experts in the key experimental and theoretical areas relevant to local-structure determination to devise a strategy for the collaborative effort required to develop a comprehensive measurement solution on the local scale. The workshop was organized by the Ceramics Division of the Materials Science and Engineering Laboratory (MSEL) at the National Institute of Standards and Technology (NIST). Participation in this meeting was by invitation only and the group size was intentionally limited to 40 to ensure a productive working environment. The final group (Fig. 1) included 10 participants from industry representing several major technological sectors, 17 from academe, 5 from government labs, 2 from the Department of Energy, and 1 from the National Science Foundation. Additionally, the meeting was attended by 14 researchers from different divisions across NIST. Representatives of the following companies participated in the meeting: Aerospace Corp., Alcatel-Lucent, ExxonMobil, Ford, General Electric, IBM, Ineos Technologies, Rigaku, and SEMATECH. Several academic attendees with industrially supported materials research programs also spoke on behalf of industry.

The program consisted of 20 oral presentations and 2 lengthy discussion sessions. The group was charged to focus on the following topics:

- Importance of Local Structure for Materials Development
- Measurement Needs and Solutions
- Existing Capabilities and Expertise
- Obstacles and Barriers
- Resources Needed
- Strategy for Integration

Specific technical areas included x-ray/neutron scattering, x-ray absorption spectroscopy, transmission electron microscopy, NMR, Raman spectroscopy, materials chemistry and physics, and solid state theory.

## 3. Highlights of Individual Presentations

The workshop was opened by the Acting Director of MSEL, Dr. Eric Amis, who gave a brief overview of the NIST mission, organizational structure, and technical activities; discussed the importance of structural knowledge for materials innovations; and emphasized the objectives of this meeting. The program included 22 oral presentations with the following titles:

1. *The need for atomic level characterization of thin films and interfaces in advanced semiconductor devices* (P. Lysaght, SEMATECH)
2. *X-ray absorption fine structure in ultra-thin films* (J. Woicik, NIST)
3. *What can we learn from thin film diffraction?* (T. Siegrist, Alcatel-Lucent)
4. *Ab initio theoretical simulations and Bayesian analyses of XANES* (J. Rehr, Univ. of Washington)
5. *Key issues in defining structure structure-properties relationships in nanoparticles and nanostructures* (S. Brock, Wayne State Univ.)
6. *The state-of-the art in neutron total scattering analysis* (T. Proffen, Los Alamos National Laboratory)
7. *Structural determination of nanoparticles by scanning transmission electron microscopy and associated techniques* (J. Liu, Univ. Missouri-St. Louis)
8. *Developing further insights into local phenomena that control time dependent performances in dielectric and piezoelectric oxides* (C. Randall, Penn State Univ. & CDS)
9. *Raman spectroscopy and electron diffraction of functional ceramics* (I. Reaney, Univ. Sheffield)
10. *How first principles studies of local structure help to explain smart material properties in oxides* (A. Rappe, Univ. Pennsylvania)
11. *The state-of-the-art in x-ray total scattering* (P. Chupas, Argonne National Laboratory)
12. *The local view and democracy* (T. Egami, Univ. Tennessee & Oak Ridge National Laboratory)
13. *Solid state NMR characterization of micro/nanoporous materials* (G. Kennedy, ExxonMobil)
14. *Simulating the coupling of chemical short-range order to polar short-range order, dielectric properties, and phase transition behavior in perovskites* (B. Burton, NIST)
15. *The measurement and interpretation of x-ray and neutron diffuse scattering* (R. Welberry, Australian National Univ.)
16. *Attaining highly reproducible EXAFS spectra: assumptions, approximations, and limitations* (F. Bridges, Univ. California—Santa Cruz)
17. *Making synchrotron and neutron radiation more relevant to industrial materials research and my experiences at General Electric* (Y. Gao, GE Global Research)
18. *RMCProfile: Studying disorder in crystalline materials* (M. Tucker, ISIS, Rutherford Appleton Lab.)
19. *Nanostructure determination and refinement from the atomic pair-distribution function* (S. Billinge, Columbia Univ.)
20. *Computational prediction of atomic- and nanoscale structure: Applications to metal alloys and hydrogen storage materials* (C. Wolverton, Northwestern Univ.)
21. *Aberration-corrected imaging of nanocrystals—current status and future prospects* (C. Hetherington, Univ. of Oxford)
22. *Electron diffraction and diffuse scattering* (R. Withers, Australian National Univ.)

The presentations were grouped in six sessions according to commonalities related to either material or measurement type. The major technical highlights are outlined below.

Patrick Lysaght (SEMATECH) described the needs for atomic level characterization of thin films and interfaces in advanced semiconductor devices. He started with an overview of the SEMATECH’s (a consortium of 16 major microelectronic companies that make up 50 % of the worldwide chip market) Front End Division. The materials needs in the semiconductor industry are driven by the progressive scaling down of microelectronic devices with the number of transistors on an integrated circuit doubling at least every 24 months. This trend requires thinner films with high dielectric constant thereby demanding new materials and/or new chip architectures. Lysaght identified the following fundamental measurement challenges related to local structure: (1) ability to probe sub-nm films introduced for doping/diffusion, (2) need to characterize *buried* layers, including sub-nm films and interfaces, composition gradients, and bonding configurations, (3) detection of small (10^12^ cm^−2^) concentrations of electrical defects, such as trap states and oxygen vacancies, and (4) difficulties with non-destructive characterization of actual devices due to limited probe sizes. Technical examples described focused on structural and chemical analyses of differently processed HfSiO-based gate stacks on Si and SiGe, and emphasized a combined use of multiple techniques, including (i) synchrotron-based x-ray photoelectron spectroscopy (XPS) and x-ray absorption spectroscopy (XAS), and (ii) transmission electron microscopy-based Z-contrast imaging and electron energy loss spectroscopy (EELS). Synchrotron experiments were described which utilized the NIST suite of beam-lines at the National Synchrotron Light Source (NSLS), including its newly commissioned X24A beamline capable of depth-resolved variable kinetic energy XPS.

Joseph Woicik (NIST) discussed applications of x-ray absorption fine structure (XAFS) to local-structure determination in thin films relevant for semiconductor microelectronics. Thin film XAFS measurements require glancing incidence to minimize the fluorescence background from the substrate, and sample spinning (or equivalent) to avoid Bragg reflections—as implemented on the NIST X23A2 beamline at the NSLS. Extended XAFS (EXAFS) probes an element-specific instantaneous distribution of interatomic distances, thus providing a powerful and often unique tool to determine the bond distances between distinct atomic pairs in alloys (e.g., In-As and Ga-As in Ga_1−x_In_x_As).

Near-edge XAFS (NEXAFS or XANES) in transition metal oxides is highly sensitive to local off-center metal ion displacements which in turn affect the properties by modifying the chemical hybridization of metal-oxygen molecular orbitals. In single crystals, the dependence of XANES on polarization of the incident radiation reflects the directions of such cation displacements. Woicik used ultra-thin epitaxial films of SrTiO_3_ on Si as an example to demonstrate that combining EXAFS, XANES, and synchrotron-based x-ray diffraction provides a very efficient means of identifying the details of structural distortions, even in very thin layers. In the case of SrTiO_3_, the epitaxial strain induces a tetragonal distortion coupled to a spontaneous electrical polarization resulting in a ferroelectric response that can be exploited for data storage. Woicik emphasized that augmenting experimental measurements with first-principles calculations is essential to discriminate among the possible structural models.

Stephanie Brock (Wayne State) provided a lively overview of the key issues in determining structure-property relationships for nanoparticles and related nanostructured materials. She used practical examples of magnetic and nonmagnetic semiconductor nanoparticles and gels to illustrate some of the principal problems that can affect properties of nanostructures, and which therefore need to be considered in the structural analyses of these materials. These problems include compositional uniformity, presence and nature of amorphous phases, structure of inter-particle interfaces, surface reconstruction, oxidation, etc. Brock indicated that such complex issues cannot be addressed using conventional powder diffraction or spectroscopy but require a combination of advanced synchrotron and electron microscopy-based techniques including those sensitive to the surface structure and chemistry.

Jimmy Liu (U. Missouri, St. Louis), who until very recently has been affiliated with Monsanto Corp., summarized the state-of-art in scanning transmission electron microscopy (STEM) of nanoparticles, while also giving an industrial perspective on the measurement needs and challenges in characterization of nanoparticles for catalyst applications. Imaging, spectroscopic, and diffraction capabilities implemented in modern STEM instruments yield quantitative information on many nanoparticle characteristics including size distributions, internal and surface atomic structure, and electronic structure of individual nanocrystals. Liu highlighted the technical capabilities of several major STEM-based techniques (e.g., high-angle annular dark field imaging, electron energy loss spectroscopy, coherent nanodiffraction) that can potentially address at least some of the measurement needs raised by Brock. The recent introduction of aberration correctors has pushed the nominal spatial resolution into the sub-angstrom range. One advantage of aberration-corrected microscopes is their ability to reveal lattice fringes regardless of nanoparticle orientation, thereby greatly facilitating phase identification and structural analysis. Despite unprecedented measurement capabilities provided by the advanced STEM instruments, obtaining an artifact-free, accurate structural solution on the nanoscale still remains a challenge. In particular, electron-induced effects on the resulting images and spectroscopic data need to be carefully considered and better understood. The grand challenge for high-spatial-resolution electron microscopy of nanoparticles from the industrial perspective is to deliver statistically representative data, and to provide quantitative comparisons of structural variations in nominally similar samples. Characteristics of interest include size and spatial distribution of nanoparticles, their shape and compositional profiles, internal defects, and surface atomic arrangements. Liu used the example of Pt nanoparticles on a Pt/C fuel cell catalyst to emphasize that different techniques, such as high-angle annular dark field imaging in STEM, wide-angle x-ray diffraction, and small-angle x-ray scattering all yield significantly different size distributions for the same sample; moreover, substantial discrepancies exist even among the results obtained using distinct TEM imaging modes. Understanding the origin of these differences is a mandatory first step to achieve a representative, accurate structural solution for nanoparticles.

Thomas Proffen (LANL) introduced the total neutron scattering method and discussed the state-of-art in relevant neutron instrumentation and data analysis capabilities. In this method, total (i.e., Bragg reflections and diffuse background) scattering in powder x-ray or neutron diffraction is Fourier transformed to obtain a real-space atomic pair-distribution function (PDF) that describes the instantaneous distribution of interatomic distances in the material. The speaker reviewed the measurement and data reduction issues involved in extracting a high quality PDF and described the technical capabilities of the NPDF diffractometer at Los Alamos—one of the few existing neutron instruments in the world optimized for PDF measurements. He highlighted recent developments in PDF data analysis software rendering it significantly more accessible to the non-expert user. Unresolved instrumental problems in neutron PDF analyses include the accurate treatment of hydrogen and corrections for inelastic scattering. On the modeling side, the challenges include lack of computer software for simultaneous refinements of PDF with other types of data (e.g., EXAFS or small-angle scattering), insufficient collaborations between the experimental and theoretical communities, and user-friendliness of computer software. The need to teach the PDF approach as a part of a routine curriculum was suggested.

Ian Reaney (U. Sheffield) outlined critical issues related to the local structure of perovskite-type materials—primary candidates for many electroceramic applications ranging from medical ultrasound to data storage to wireless communications—and showed how Raman spectroscopy can be used to probe structural details in these materials. Examples included combined Raman and electron diffraction studies of defect chemistry and the effects of dopants on phase transitions in BaTiO_3_, phase relations in industrially-relevant Pb(Zr,Ti)O_3_, and structure-property relations in commercial microwave dielectrics. Raman spectroscopy is highly sensitive to order-disorder behavior in both pure and doped BaTiO_3_ suggesting that this technique may be used to identify the charge compensation mechanisms for doping in BaTiO_3_. Raman spectroscopy also effectively reveals local structural changes in other perovskite ferroelectrics/dielectrics. Reaney emphasized the importance of multiple-technique studies that combine Raman with other methods to establish the structural origin of various Raman features. At present, Raman spectroscopy provides only qualitative information on the local structure of perovskites because of the lack of appropriate underlying theory for disordered systems.

Clive Randall (Penn State), who heads the Center for Dielectric Studies (a consortium of over 20 companies), continued the topic of electroceramics and presented an industrial perspective on local phenomena which control the time-dependent performance of dielectric and piezoelectric materials. Modern commercial actuators such as those used for fuel injection consist of many (≈350) active piezoelectric layers. The primary reliability issues in these devices are related to interlayer diffusion, reactions, and electromigration. Similar to semiconductor devices, characterization challenges involve analyses of buried layers and interfaces; however, relatively large layer thicknesses (sub-micron range) in electroceramic systems limit the usefulness of grazing incidence and variable energy measurement techniques which enable non-destructive depth profiling in microelectronic heterostructures. Frequently, the choice of measurement methods for determination of local structure and chemistry in multi-layered electroceramic architectures is limited to cross-sectional electron microscopy and associated spectroscopic tools (e.g., EELS). Yet, use of other methods, such as confocal Raman spectroscopy, needs to be further explored. Another example of reliability issues is related to an amorphous-to-crystalline transformation in multilayered electrolytic capacitors, because crystallization can be detrimental for device performance. Randall emphasized the potential usefulness of fluctuation microscopy as implemented in STEM—a relatively novel technique for characterization of medium-range order—to identify local crystalline regions in amorphous materials. A different set of measurement problems is posed by the needs of the multilayered ceramic capacitor (MLCC) industry because the ability to understand local failure mechanisms is a central issue for this type of electroceramic device. Examples of combining voltage-contrast scanning electron microscopy, used to identify potential failure sites, with microprobe-based complex impedance measurements at the same locations were presented. The speaker summarized the principal industrial measurement needs and challenges for reliability-related issues in electro-ceramic devices as: (1) characterization of local structure and chemistry in amorphous materials, buried interfaces, and grain boundaries; and (2) techniques for establishing structure-property relations for fast-breakdown measurements.

Andrew Rappe (UPenn) described recent progress in *ab initio* calculations of local structure and properties of perovskite ferroelectrics. The perovskite ABO_3_ structure can accommodate nearly any chemical element within its highly flexible network of [BO_6_] octahedra, thereby providing a rich palette for materials discovery. One goal of the theoretical work is to extract general rules that relate composition and properties. Chemistry affects the properties of perovskites through various (often interrelated) types of structural distortions which can exhibit correlations over length-scales ranging from atomic to macroscopic. Modern density functional theory (DFT) calculations allow precise analyses of the atomic response to the local environment, and can accurately reproduce the main features of the experimental PDF results in a variety of perovskite-like systems. The speaker presented several examples demonstrating that knowledge of local structure is crucial for understanding perovskite ferroelectric solid solutions. In particular, knowledge of cation off-center displacements in perovskites, which depend on the local chemical environment, enables the prediction of Curie temperature, relaxor dispersion, and the location of morphotropic phase boundaries—all of which have major impacts on the functional performance of ferroelectrics. First-principles calculations add new crystal chemical concepts and reveal interplay of various interatomic interactions. Rappe emphasized that combining experimental data and first principles calculations yields more accurate composition-structure-property relations, thereby facilitating the rational design of new materials.

Peter Chupas (ANL) reported on the current state of PDF measurements using x-ray radiation at the high-energy facilities (1-ID, 11-ID) of the Advanced Photon Source in Argonne. Combining high-energy (57 keV to 120 keV) incident x-ray radiation with efficient data collection using advanced area detectors enables rapid (up to 30 Hz) high-resolution PDF measurements thereby providing unprecedented opportunities for *in situ* time-resolved studies of local structure during chemical/phase reactions in nanostructured materials. For example, this method was applied to measure the kinetics of Pt-catalyst nanoparticle formation on TiO_2_. X-ray PDF measurements can also be effectively used to study hydrogen location in certain hydrogen storage materials, such as porous coordination frameworks. The speaker presented experimental data showing that x-ray PDF was sensitive enough to identify H_2_-framework interactions in hydrogen-loaded Mn_3_[Co(CN)_6_]_2_. Future measurement challenges for x-ray PDF include implementation of simultaneous multiple-technique (e.g., PDF, SAXS) measurements, currently hindered by the lack of appropriate high-energy area detectors, and chemically-resolved PDF measurements involving resonant scattering.

Frank Bridges (UCSC) focused on metrological aspects of EXAFS and comparisons of this method with other techniques. He emphasized that highly reproducible EXAFS data can be attained by using sufficiently uniform samples and an unfocused beamline. In cases (e.g., small samples) where a focused beamline is preferred, additional special care must be exercised to ensure beam/sample uniformity. Despite high x-ray fluxes available at modern synchrotron sources, the inadequate performance of the existing fluorescence detectors still limits both the quality and throughput of EXAFS measurements on dilute and thin-film samples. Bridges suggested that increasing the number of elements in fluorescence detectors will significantly improve their dynamic range. The speaker presented a thorough discussion of Debye-Waller (D-W) factors, which describe peak widths in atomic PDF as determined by EXAFS and other methods. For example, EXAFS and x-ray/neutron PDF both probe instantaneous distributions of interatomic distances and should provide similar values of D-W factors; yet, some cases of disagreement were encountered, requiring a more detailed comparison of these two techniques.

Gordon Kennedy (ExxonMobil) described the use of solid-state nuclear magnetic resonance (NMR) for characterization of local structure in mesoporous materials. The resonance shifts observed in NMR are very sensitive to the local coordination environment of the excited nuclei, whereas the measured absorption is directly proportional to the mole fraction of these atoms in the sample. This technique is ideally suited for characterization of micro/nanoporous materials as they contain many NMR-active isotopes. NMR provides information on structural short-range order and can directly probe the distribution of both framework elements and acid sites. NMR results can be readily combined with diffraction data providing useful constraints in local-structure models. The capabilities of various NMR modes and configurations were discussed. Current challenges include development work on signal enhancement and detection methods to improve resolution and sensitivity of this method.

Benjamin Burton (NIST) presented the current state-of-the-art in first-principles simulations of systems with combined chemical and polar short-range order. Notable examples of industrially-relevant materials having properties critically dependent on this type of local phenomena include relaxor ferroelectrics and shape-memory alloys. The first-principles effective Hamiltonian is a powerful length-scale-bridging tool for enabling simulations of ≈300 000-atom systems. Molecular-dynamic simulations of first-principles Hamiltonians effectively revealed the nature of correlations between chemical and polar nanoscale order in several classical relaxor ferroelectric systems. The shape of the neutron diffuse scattering calculated for simulated nanostructures agreed qualitatively with the available experimental data. Burton stressed that theoretical modeling of nanostructured materials requires building nanoscale models—a capability readily provided by an effective Hamiltonian technique. The principal obstacles for a broad application of this method include the lack of automation for deriving an effective Hamiltonian in a given system, and the need for a computational toolbox to calculate bulk properties and diffuse scattering, and to identify correlated regions in the simulated models.

Simon Billinge (Columbia University) articulated the “nanostructure problem” and emphasized the absence of robust comprehensive measurement solutions. The state-of-the-art in nanostructure solution and refinement approaches using x-ray/neutron PDF was discussed through case studies of various systems. The most important obstacle is the inability of any single measurement technique to constrain a unique structural solution; therefore, integration of information from multiple methods is essential. The main unresolved issues include obtaining data from several techniques (frequently requiring highly specialized equipment and expertise) on identical or similar samples within a relatively short time, and subsequent quantitative integration of these data in a combined structural analysis; the latter issue still remains a fundamental research problem. The speaker suggested that complex modeling of nanostructured materials combining experiment and theory in a coherent computational network would be a large step toward solving the nanostructure problem. Billinge further proposed a complex modeling “institute” that would provide “a clearing house” to match synthetic scientists with experts in measurements and theoretical calculations, and which would develop and host computer software for complex modeling in an open-source framework while providing necessary education and training.

John Rehr (U. Washington) reviewed recent progress in theoretical simulations of x-ray absorption near edge structure (XANES). This is a powerful local-structure probe complementary to EXAFS which measures electronic structure (i.e., local density of states, valence, etc.) but is also sensitive to the 3-D details of local atomic configurations. The speaker discussed recent advances in the development of parameter-free *ab initio* theory for calculating x-ray absorption spectra, which is a necessary prerequisite for the use of XANES as a fully quantitative technique. Structural analyses using XANES are hindered by the large number of parameters required to fit experimental data, making conventional least-squares fits ill-conditioned. The problem can be alleviated using modern Bayesian statistical analyses based on conditional probabilities and *a priori* knowledge of the system. Recently, such Bayesian analyses that enable combined fittings of XANES and EXAFS data were implemented as an add-on to public-domain software. Rehr expressed confidence that most goals to achieve quantitative local-structure analyses using fine structure in x-ray absorption spectra are either addressed or within sight.

Yan Gao (General Electric) presented an overview of materials characterization at GE using synchrotron and neutron radiation and gave his perspective on making these facilities more relevant to industrial research and problem-solving. Gao indicated that most engineering applications require high-energy x-rays and mentioned the need for the following high-energy beamline capabilities: non-destructive residual stress measurements, white-beam strain mapping, *in situ* high-temperature mechanical testing, and high-temperature x-ray diffraction. Some of the limitations mentioned include the absence of mild-energy XAFS beamlines for measuring Mg, Al, and Si K-edges, and adapting x-ray imaging to “real-world problems”. Gao stressed the importance of bridging the existing gap between the “people who have the tools” and “people who have the problems” and underlined the need for a balance between the “best for science” and “most useful for characterization” in the planning and development of synchrotron and neutron user facilities. (In the ensuing discussion, J. Woicik pointed out that the NIST’s X24A beamline at the NSLS will address the need for XAFS of Mg, Al, and Si.)

Mathew Tucker (ISIS) discussed applications of the Reverse Monte Carlo (RMC) method to local-structure determinations in crystalline materials. In the RMC approach, the atoms arranged in a large cell with periodic boundary conditions are allowed to move according to the Monte Carlo algorithm until a match between the calculated and experimental data is obtained. The RMCProfile software, developed at ISIS, can simultaneously fit total x-ray/neutron scattering intensity, real-space PDF, and Bragg profile under various structural restraints thereby enabling consistent refinements of both local and average atomic structures in the same model. Recently, this software was extended to incorporate element-specific EXAFS in a combined fit with otherwise chemically unresolved total scattering data; this development was a result of the NIST-ISIS collaboration. Rigorous methodology for treating multiple datasets has yet to be developed; for example, one unresolved but critical question concerns weights assigned to different types of data being fit. Tucker noted that RMCProfile provides an excellent platform for exploring multiple-technique structural refinements because it can incorporate any type of experimental data which can be calculated for a large-size model.

Chris Wolverton (Northwestern Univ.) reviewed advances in theoretical predictions of atomic and nanoscale structures as applied to metal alloys and hydrogen-storage materials. Existing structure-prediction methods based on DFT calculations were discussed and their capabilities, as applied to the search for an optimized hydrogen-storage material, were compared. The cluster expansion method was shown to be an effective tool to bridge the scales of first-principles quantum-mechanical total energy calculations and thermodynamic or kinetic Monte Carlo simulations, thereby enabling accurate modeling of structure and properties for large (100 000 atoms) atomic ensembles. This approach (with coherency strain taken into account) can be used to predict a variety of alloy characteristics including low-temperature long-range order, precipitate shape, order/disorder transition temperature, high-temperature short-range order in solid solutions, and various thermodynamic properties. Furthermore, first-principles kinetic Monte Carlo simulations provide accurate information on the nanostructure evolution. As in the cases described by Burton, the resulting models can be used to predict diffuse scattering arising from short-range order. Wolverton also commented on the inverse problem of determining a real-space structure from pair-distribution function/diffuse scattering. The relation is unique only for systems having a Hamiltonian described strictly by the pair-wise inter-atomic interactions; otherwise, pair-correlations do not completely determine higher-order correlations.

Crispin Hetherington (Univ. Oxford) discussed the current state of the aberration-corrected TEM and demonstrated the capabilities of this advanced instrumentation for studies of catalyst nanoparticles and grain boundaries in ceramics. The ability to directly interpret high-resolution TEM (HRTEM) images is limited by the aberrations of the image-forming lens. The speaker described major strategies for aberration correction (direct hardware correction, indirect computational compensation, and diffractive imaging) and then focused on the characteristics of the double corrector (i.e., probe- and imaging-probing lenses) optics as implemented on the JEOL2200FS microscope in Oxford. Direct aberration correction drastically reduces delocalization effects in HRTEM thereby greatly facilitating image interpretations, especially for aperiodic features such as surfaces and interfaces. Combining direct and indirect corrections, such as a restoration of the complex exit wavefunction, further increases the amount of information that can be retrieved from the HRTEM images. Aberration-corrected TEM offers enhanced capabilities for *in situ* studies due to the larger space available within the objective lens. Double correction, while enabling aberration-corrected imaging in both HRTEM and STEM modes, offers the additional possibility of confocal STEM for three-dimensional imaging and analyses.

Ray Withers (Australian National Univ.) highlighted the use of electron diffuse scattering as a probe for local atomic order. In contrast to powder diffraction techniques, electron diffraction in TEM can probe individual small single crystals and, by varying crystal orientation, yield a three-dimensional distribution of diffracted intensities. Additionally, electrons interact with matter ≈10 000 times more strongly than either x-rays or neutrons, thus making electron diffraction highly sensitive to the details of local atomic order. Many complex materials feature structured diffuse scattering confined to a well-defined geometric locus. The shape of this locus reflects the relations among the atomic correlation parameters, the character of the intensity distribution along the locus indicates the nature (displacive or chemical) of the local order, and the extinction conditions for the diffuse intensities encode relations among the directions/magnitudes of atomic displacements and site occupancies. Withers described a rigorous approach for interpreting electron diffuse scattering in terms of local displacive/chemical order and illustrated the power of this technique using several complex material systems.

## 4. Discussion Summary

### 4.1 Measurement Needs, Existing Obstacles, and Critical Resources

During the two substantial “round-table” discussion sessions of the workshop, the participants identified the following *highest and equal priority* industrial measurement needs for local structure determination:

- Quantitative *non-destructive* measurements of buried layers (crystalline or amorphous) and interfaces (heterophase and grain boundaries), composition gradients, low concentrations of dopants, and electrically-active defects;
- Methods that will produce *representative* data on relevant structural, morphological, and compositional characteristics of nanostructured materials suitable for quantitative comparison of nominally similar samples; quantitative characterization of *heterogeneous* and *multiphase* nanostructured samples; and
- *In situ* measurements providing interpretable structural and chemical information under actual material/device operating conditions.

The first need is driven primarily by the microelectronic and electroceramic industries, whereas the second challenge is dictated by applications of nanoparticles and related materials. *In situ* measurements are critical for all industrially-relevant systems because “real-world” operating conditions cannot be neglected.

Addressing these challenges is critically dependent on advances in instrumentation. Many of these measurements require spatially-localized, high-energy, high-brightness probes typically available only at the national synchrotron or neutron facilities. The new synchrotron (e.g., NSLS-II) and neutron (e.g., SNS) sources will provide suitable incident beams; however, taking full advantage of these high-cost capabilities requires significant effort in developing end-station instrumentation and relevant, user-friendly computer software. The same situation exists for the state-of-the-art aberration-corrected electron microscopes. In many cases, atomic positions need to be known with a precision of 1 picometer (10^−12^ m) or better which is beyond the current resolution limits of even aberration-corrected TEM. One approach to address this problem involves development of quantitative image-interpretation methods based on physics-based models for electron-object interactions (e.g., statistical parameter estimation technique). Many participants emphasized the need for improved detectors to match the high fluxes of incident radiation and enable efficient data collection, including the ability to collect multiple types of data (e.g., x-ray PDF and SAXS) in a single experiment. In some measurements (e.g., diffuse x-ray scattering), lack of efficient discrimination of the dominant signal from the underlying average structure remains a limiting issue.

Comprehensive characterization of nanoparticles or nanoporous materials requires quantitative measurement of a large set of statistically distributed parameters describing shape, size, internal structure, chemistry, etc. that usually involve use of multiple techniques. Furthermore, many materials of industrial relevance are heterogeneous, multiphase systems. One major barrier to addressing these issues is the lack of established methodology for parameterization of the problem and self-consistent interpretation of multiple types of data—a prerequisite for establishing the critical link between the characteristics of individual nanoparticles as probed with spatially-resolved methods, and the statistical features of nanoparticle samples obtained from powder-averaging techniques. The development of such methodology is further hindered by the paucity of systematic comparison-studies of the available measurement techniques using reference samples. In addition, studies of the dynamic behavior of nanoparticles require tools with high temporal resolution at femtosecond (10^−15^ s) to attosecond (10^−18^ s) time scales. Examples of emerging ultrafast probes include 4-D electron imaging and time-resolved XAS.

A questionnaire was distributed among the participants to gather their views on the most important obstacles to solving the nanostructure problem and the most critical resources needed to overcome these obstacles. The responses are summarized in Table 1. All participants—industrial, government agency, and academic—acknowledged that the present state of local structure measurements is inadequate for addressing not just industrial but even many fundamental research needs. The complex, multifaceted nature of the nanostructure problem presents several challenges that must be addressed simultaneously. The *highest and equal-priority* instrumental, data analyses, and organization resources needed for solving this problem include:

- Instrumentation for non-destructive probing of buried features and *in situ* local structure measurements with high spatial and high temporal (for dynamic studies) resolutions; improved detector systems for matching the capabilities of modern synchrotron, neutron, and electron radiation sources;
- Methodology and computer software for combining inputs from multiple techniques and theory in a global complex modeling framework;
- Theoretical tools and software for modeling nanoscale structures and their measurement responses, including those attainable using bench-top instrumentation; and
- A network for dynamic linkage between industrial/academic users and measurement experts.

The participants reached a consensus that a comparison-study of the principal local-structure measurement techniques using deliberately-selected nanoparticle samples is warranted as a critical step toward the multiple-technique integration. The study would involve total scattering PDF, small-angle scattering, EXAFS, and TEM/STEM. NIST will organize and coordinate this inter-laboratory activity.

### 4.2 Strategy for Solving the Nanostructure Problem

The participants unanimously agreed that solving the nanostructure problem—an ultimate *frontier* in materials characterization—necessitates a coordinated interdisciplinary effort that transcends the existing capabilities of any single institution, including national laboratories, centers, and user facilities. Such an ambitious activity will require close cooperation among experts in local structure measurements (utilizing x-ray, neutron, electron, and optical probes), solid state theory, materials synthesis, device applications, information technology, computer programming, etc.

**Fig. 2:**
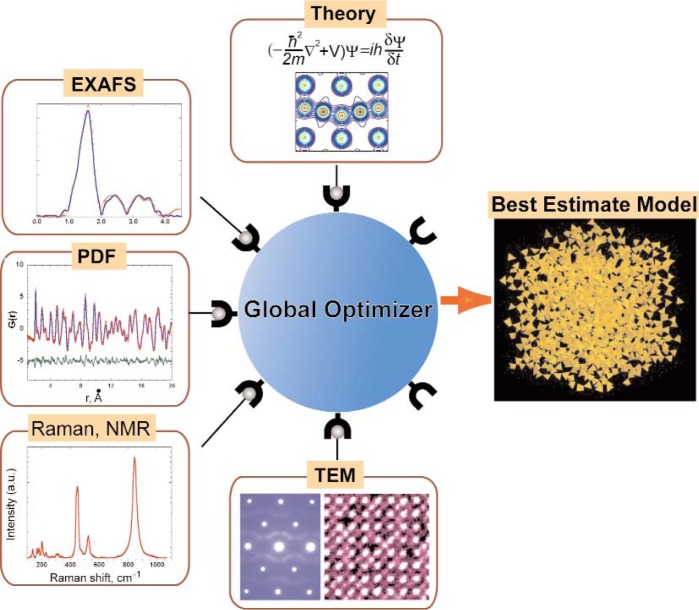
A schematic representation of the complex modeling idea: integration of combined inputs from multiple experimental techniques and theory into the global optimization framework [Billinge and Levin, unpublished]. In this concept, the structural model evolves toward the correct solution as more data is provided for the fit.

The discussions converged on an *institute* dedicated to local structure determination as the most viable organizational platform for successfully addressing the nanostructure problem. The proposed “institute” would provide an intellectual infrastructure for local structure determination by (1) developing and maintaining relevant computer software integrated in an open-source global optimization framework (Fig. 2), (2) connecting industrial and academic users with experts in measurement techniques, (3) developing and maintaining pertinent databases, and (4) providing necessary education and training. The institute, whether “virtual” or “real”, was envisioned to have beam time allocated at national synchrotron, neutron, and electron microscopy facilities to enable a synergistic, coordinated use of these facilities for solving local structure problems. Clearly, securing significant and sustained government funding through an *interagency initiative* will be critical to enable this collaborative project. The cost of the proposed institute, while substantial, will represent only a small fraction of the taxpayer investment in the existing national user facilities and nanotechnology centers; moreover, the institute will leverage investments in these facilities from other sources interested in solving the nanostructure problem.

## 5. Feedback From Workshop Participants

Forms requesting anonymous feedback on the workshop were distributed to all participants. Attendees were overwhelmingly positive in indicating that the workshop was worth their time and that another should be held. Selected written comments on the three critical issues pertinent to solving the nanostructure problem are quoted below:

*Importance of solving “the nanostructure problem” for future technological developments*
  - Key material properties rely on structures that are different from the average, and therefore nearly impossible to study.
  - Clearly very important both for manufacturing control in industry and structure-properties relationship determination in research.
  - Nanostructured materials will be pervasive in society, therefore, understanding the problem and having solutions and methods to address it will be crucial.
*Contribution of this workshop to solving the “nanostructure problem”*
  - Excellent workshop because there was an excellent cross-section of the community who can make recommendations that have broader validity.
  - The workshop provided a state-of-the-art summary of current issues, and identified future issues and challenges.
  - The workshop brought together people with problems and those with techniques with the potential to solve them; defined needs of the community.
*The most effective role for NIST in solving “the nanostructure problem”*
  - Interface to industrial problems, fund and work on developing standards for quality of nanostructure solution, provide leadership on directing what kinds of nanostructure problems should be addressed to benefit industry, host quality-assured databases of data and metadata, run round robin tests and make local-structure standard samples;
  - Act as a coordinator working with selected experts in various techniques to solve some key problems such as structure of various types of nanoparticles.
  - Provide reference materials for structure-models validation; support future development of characterization methods, data analyses, and modeling activities; coordinate/lead integration effort; enhance opportunities for education and training in characterization of nanoparticles;
  - Manage this complex interdisciplinary, multi-institutional effort; become the point of contact for the community interested in working on the problem;
  - Connecting theory and experiment; publishing standards for how data should be processed;
  - Supporting and coordinating the cross-collaboration of the groups;
  - Could serve as a center for local-structure nanoparticle studies, perhaps to hold a series of yearly meetings.

## Figures and Tables

**Table 1:** Summary of major obstacles to solving the nanostructure problem and critical resources needed, as identified by the workshop participants

Obstacles	Critical Resources
*Instrumentation*	
• Development of non-destructive probe techniques with high spatial, high temporal resolution	• Instrumentation for probing buried features with high spatial and temporal resolution
• Development of end-station instrumentation, especially detector systems, to realize the capabilities provided by the modern synchrotron, neutron, and electron sources	• Instrumentation for in operando measurements
	• Funding for development of better detectors
*Data analysis and interpretation*	
• Development of easy-to-access bench-top techniques (e.g., Raman, NMR) into quantitative probes of local structure	• Theoretical tools, including user-friendly software for modeling of nanoscale structures and prediction of their spectroscopic responses
• Development of efficient automated theoretical methods and robust, user-friendly real-space computer codes for calculating electronic, vibrational, and magnetic structure, and modeling the measurement responses (e.g., XANES, Raman)	• Comparison of available measurement techniques including round-robin tests on reference materials
• Development of structure-solving approaches using multiple experimental techniques and theory	• Methodology and user-friendly software for combining inputs from multiple techniques in a global complex modeling framework
*Organizational*	
• Fostering interactions between materials researchers and experts in various measurement methods, and between experimentalists and theorists to facilitate interpretation and analysis of experimental data	• Databases of characterized structures, available measurement methods, software
	• Dynamic network linking industrial and academic users with measurement experts
• Education and training	• Fast access to a wide range of techniques
	• Funding for collaborative research
	• Improved interactions between different branches of the community, regular meetings, Gordon Research Conferences on local structure measurements

## List of Acronyms

DFT: Density Functional Theory
EELS: Electron Energy Loss Spectroscopy
HRTEM: High Resolution Transmission Electron Microscopy
MLCC: Multilayered Ceramic Capacitor
NEXAFS (or XANES): Near-Edge X-ray Absorption Fine Structure
NMR: Nuclear Magnetic Resonance
NSLS: National Synchrotron Light Source
PDF: Pair-Distribution Function
RMC: Reverse Monte Carlo
SAXS: Small-Angle X-ray Scattering
SNS: Spallation Neutron Source
STEM: Scanning Transmission Electron Microscopy
TEM: Transmission Electron Microscopy
XAFS: X-ray Absorption Fine Structure
XAS: X-ray Absorption Spectroscopy
XPS: X-ray Photoelectron Spectroscopy

**List of Participants**

Andrew Allen (NIST)
Eric Amis (NIST)
Ian Anderson (NIST)
Simon Billinge (Columbia Univ.)
Frank Bridges (Univ. California, Santa Cruz)
Stephanie Brock (Wayne State Univ.)
Benjamin Burton (NIST)
Peter Chupas (Argonne National Laboratory)
Eric Cockayne (NIST)
Robert Cook (NIST)
Takeshi Egami (Univ. Tennessee & Oak Ridge National Laboratory)
Lixin Fan (Rigaku)
Chris Fischer (MIT)
Anatoly Frenkel (Yeshiva Univ.)
Yan Gao (GE Global Research)
Crispin Hetherington (Univ. Oxford)
James Kaduk (Ineos Technologies)
Debra Kaiser (NIST)
Stanislav Kamba (Inst. Physics, Czech Republic)
Gordon Kennedy (ExxonMobil)
Helen Kerch (Department of Energy)
Rajiv Kohli (Aerospace Corp.)
Igor Levin (NIST)
Zachary Levine (NIST)
Jingyue Liu (Univ. Missouri)
Michael Lufaso (Univ. North Florida)
Patrick Lysaght (SEMATECH)
Lynnette Madsen (National Science Foundation)
Sara Mason (NIST)
Wilfried Mortier (ExxonMobil)
Conal Murray (IBM)
Thomas Proffen (Los Alamos National Laboratory)
Clive Randall (Penn State Univ.)
Andrew Rappe (UPenn)
Bruce Ravel (NIST)
Ian Reaney (Univ. Sheffield)
John Rehr (Univ. Washington)
Robert Roth (NIST)
Eric Shirley (NIST)
Don Siegel (Ford)
Theo Siegrist (Alcatel-Lucent)
Matthew Tucker (ISIS, UK)
Terrell Vanderah (NIST)
Mark Vaudin (NIST)
Thomas Welberry (Australian National Univ.)
Lane Wilson (DOE)
Ray Withers (Australian National Univ.)
Joseph Woicik (NIST)
Chris Wolverton (Northwestern University)
Patrick Woodward (Ohio State Univ.)
